# Right ventricular rupture and tamponade caused by malposition of the Avalon cannula for venovenous extracorporeal membrane oxygenation

**DOI:** 10.1186/1749-8090-7-36

**Published:** 2012-04-20

**Authors:** Hitoshi Hirose, Kentaro Yamane, Gregary Marhefka, Nicholas Cavarocchi

**Affiliations:** 1 Department of Surgery, Division of Cardiothoracic Surgery, Thomas Jefferson University, Philadelphia, PA, USA; 2 Department of Medicine, Division of Cardiology, Thomas Jefferson University, Philadelphia, PA, USA

**Keywords:** ECMO, Tamponade, Surgery, Pneumonia, Respiratory failure

## Abstract

Placement of the Avalon Elite bicaval dual lumen cannula for venovenous extracorporeal membrane oxygenation (VV-ECMO) via the internal jugular vein requires precise positioning of the cannula tip in the inferior vena cava with echocardiography or fluoroscopy guidance. Correct guidewire placement is clearly the key first step in assuring proper advancement of the cannula. We report a case of unexpected wire migration into the right ventricle at the time of final cannula advancement, resulting in right ventricular rupture and tamponade. Transesophageal echocardiography is an important monitoring modality for appropriate placement of the VV-ECMO guidewire and Avalon cannula, and in particular, for early identification of potential complications.

## Background

**Figure 1:**
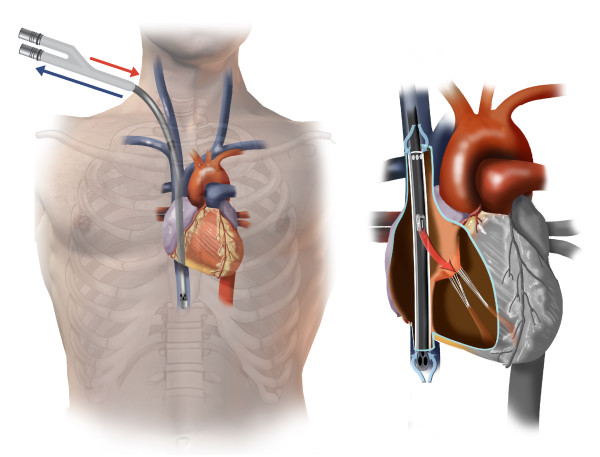
**Illustration of Avalon Elite bicaval dual lumen cannula in correct position**.

The Avalon Elite bicaval dual lumen cannula (Avalon Laboratories, Rancho Dominguez, CA) has been used for venovenous extracorporeal oxygenation (VV-ECMO) [1,2]. The cannula consisted of 2 lumens: one lumen allows the deoxygenated blood to drain from the distal and proximal ports, from the inferior vena cava (IVC) and the superior vena cava (SVC), respectively; and a second lumen allows the oxygenated blood to return from the external pump to the right atrium directed toward the tricuspid valve (Figure 1). We have used the Avalon cannula for adult VV-ECMO in salvageable patients with severe refractory adult respiratory distress syndrome (ARDS) since 2009. We have performed VV-ECMO in 4 patients specifically using the Avalon cannula system since then, with successful weaning in all 4 patients. We describe one patient who developed right ventricular rupture and acute cardiac tamponade at the time of cannula insertion.

## Case presentation

**Figure 2:**
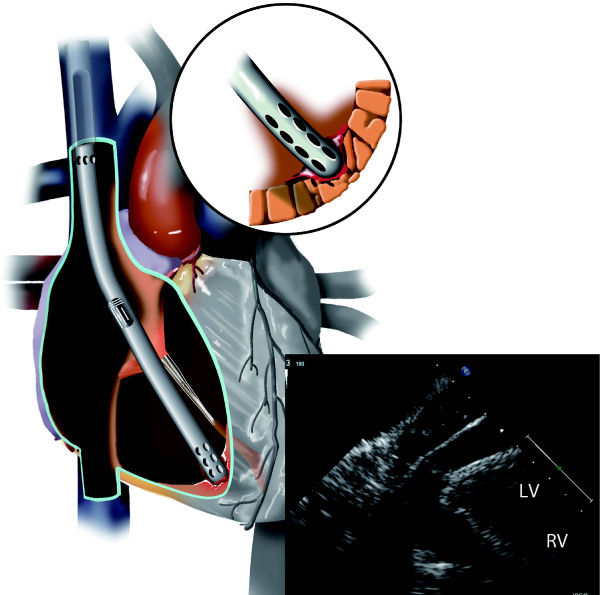
**Illustration of the right ventricular injury by Avalon cannula and echocardiography evidence of pericardial tamponade**. LV: left ventricle. RV: right ventricle.

A 53 year old female without significant past medical history developed severe viral pneumonia, with rapid, progressive deterioration in her respiratory status. She developed ARDS and mechanical ventilatory management using ARDS protocol were unable to maintain adequate oxygenation. As a result, bedside VV-ECMO was planned. Transesophageal echocardiography (TEE) was performed to visualize proper positioning of the guidewire and cannula. Using the Seldinger technique, the right internal jugular vein was accessed and a guide wire was placed. Placement of the guidewire into the IVC proved difficult due to repeated migration of the guidewire into the right ventricle. After multiple attempts, the guidewire was visualized to course properly from the SVC to the IVC. After a bolus dose of 5000 units of intravenous heparin was given, the right internal jugular venous access site was dilated. Just as the final dilatation was completed and upon dilator exchange with simultaneous advancement of the 23 French Avalon cannula, TEE lost visualization of the guidewire. Multiple premature ventricular beats were noted and immediately, a new, rapidly enlarging pericardial effusion was detected (Figure 2). Emergent preparations were made for bedside surgical decompression of the pericardial space. Quickly the patient lost blood pressure from acute cardiac tamponade. The Avalon cannula was immediately clamped at the end but not removed. A emergent subxiphoid pericardial window was performed, resulting in drainage of venous blood and restoration of blood pressure. Transfusion was initiated and the patient was emergently transported to the operating room for surgical exploration. The Avalon cannula was found to have perforated the apex of the right ventricle. The injury was repaired primarily and the Avalon cannula was repositioned toward the IVC again by TEE with additional direct manipulation. VV-ECMO was initiated and the oxygenation improved. Due to excessive coagulopathies, the sternum was left open but was closed on postoperative day 2. From that point, she remained free from any cardiac or infectious complications and her pulmonary condition slowly improved. She was successfully weaned from VV-ECMO on postoperative day 9 and was discharged home on postoperative day 24 without the need for home oxygen. She regained full physical functions at home and recovered normal pulmonary function by 3 months following discharge from the hospital.

## Discussion

Correct positioning of Avalon cannula during placement of VV-ECMO is crucial for avoiding complications and to ensure effective oxygenation. The drainage ports of the Avalon cannula should be in the SVC and IVC optimizing removal of deoxygenated blood, with the infusion port in the mid right atrium directed toward the tricuspid valve optimizing delivery of oxygenated blood directly toward the right ventricle and diseased lung. TEE can be an effective modality in confirming proper positioning of cannula. Once the cannula is placed in the correct position, VV-ECMO using Avalon cannula from the right internal jugular access has significant advantage over conventional femoral-femoral or femoral-jugular VV-ECMO. Since Avalon cannula does not require second femoral drainage cannula, the patient is able to sit up, to receive pulmonary toilet and to participate in limited physical therapy, all of which are important in critically ill patients with ARDS.

However, placement of the Avalon cannula may be difficult and has the potential to lead into life threatening complications as described in this report. The guide-wire may not easily advance into the IVC and could migrate across the tricuspid valve and into the right ventricle. This may be caused by the presence of a large Eustachian valve in the right atrium, prohibiting the guidewire passing into the IVC. The wire may curl at the Eustachian valve or in the right atrium and prolapse into the right ventricle. Guidewire malposition should be suspected if there are runs of the premature ventricular complex noted, which should prompt the surgeon to withdraw the guidewire. If the guidewire subsequently migrates to the right ventricle undetected, it could lead to right ventricular rupture from the guidewire, the dilators, or the cannula itself. Avalon cannula placement requires either echocardiographic or fluoroscopic guidance. We use bedside TEE for Avalon cannula placement because most patients requiring VV-ECMO are not stable for transportation to a catheterization or interventional radiology laboratory. Fluoroscopic guidance may however provide better confirmation and continued surveillance of proper guidewire and cannula positioning.

In our patient, we suspect that during the dilator exchanges, the guidewire may have been accidentally pulled back from its originally confirmed position in the IVC and/or prolapsed into the right ventricle just as the final Avalon cannula was being advanced, which occurred simultaneously with loss of visualization of the guidewire within the IVC. The complication was quickly recognized as a new pericardial effusion was appreciated, at the same time as attempting to revisualize the guidewire and cannula position. Subsequently cardiac tamponade was recognized before patient lost her blood pressure, and emergent preparation for pericardial window was performed at the bedside without delay, saving the patient's life.

## Conclusions

TEE is an important bedside imaging modality to guide proper placement of the Avalon cannula for VV-ECMO via the internal jugular vein, and can be essential in the early detection of related complications.

## Consent

Written consent was obtained from the patient for publication of the case report and accompanying images. A copy of the written consent is available for review by the Editor-in-Chief.

## Abbreviations

ARDS: Adult respiratory distress syndrome; ECMO: Extracorporeal membrane oxygenation; IVC: Inferior vena cava; SVC: Superior vena cava; TEE: Transesophageal echocardiography; VV-ECMO: Venovenous extracorporeal membrane oxygenation.

## Competing interests

The authors declare that they have no competing interests.

## Authors' contributions

HH: conception and design, analyses of data, drafting the manuscript and final approval. KY: analyses of data, drafting the manuscript and final approval. GDM: analyses of data, drafting the manuscript and final approval. NC: analyses of data, drafting the manuscript and final approval. All authors read and approved the final manuscripts.
